# Accuracy of conization procedure for predicting pathological parameters of radical hysterectomy in stage Ia2–Ib1 (≤2 cm) cervical cancer

**DOI:** 10.1038/srep25992

**Published:** 2016-05-16

**Authors:** Huimin Bai, Dongyan Cao, Fang Yuan, Huilan Wang, Meizhu Xiao, Jie Chen, Quancai Cui, Keng Shen, Zhenyu Zhang

**Affiliations:** 1 Department of Obstetrics and Gynecology, Beijing Chao-Yang Hospital affiliated with China Capital Medical University, Beijing, China; 2 Department of Obstetrics and Gynecology, Peking Union Medical College Hospital, Chinese Academy of Medical Sciences & Peking Union Medical College, Beijing, China; 3 Department of Obstetrics and Gynecology, the Affiliated Hospital of Medical College Qingdao University, Qingdao, China; 4 Department of Obstetrics and Gynecology, the Second Hospital of Hebei Medical University, Shijiazhuang, China; 5 Department of Pathology, Peking Union Medical College Hospital, Chinese Academy of Medical Sciences & Peking Union Medical College, Beijing, China

## Abstract

The accuracy of conization for the prediction of radical hysterectomy (RH) pathological variables in patients with stage Ia2 to Ib1 (≤2 cm) cervical cancer was retrospectively evaluated in the present study. Endocervical or deep resection margin (RM) involvement in the conization specimens was found to be independently associated with residual disease in the hysterectomy specimens (P < 0.001, = 0.003, respectively). When a tumor width of >20 mm in the final RH pathology analysis was predicted by a tumor width of >2 mm or involvement of endocervical or deep RMs in the conization specimens, the sensitivity and negative predictive value (NPV) of conization were 98.2% and 95.2%, respectively. In addition, when deep stromal invasion in the final RH pathology analysis was predicted by deep stromal invasion or involvement of the endocervical or deep RMs in the conization specimens, the sensitivity and NPV of conization were 98.4% and 95.8%, respectively. The sensitivity and NPV of this prediction model for identifying LVSI in the final RH pathology analysis were both 100%. These findings suggest that conization variables and endocervical and deep resection margin statuses can be analyzed to effectively predict RH pathological parameters.

With the development of improved screening for cervical cancer, an increasing number of cases are being diagnosed at early stages^1^,^2^. The standard surgical management for stage Ia2 to Ib1 cervical cancer is radical hysterectomy (RH) with pelvic with or without paraaortic lymphadenectomy^3^. However, the issue of parametrectomy has been a subject of controversy in women with small-volume tumors. For women with a largest tumor diameter of ≤2 cm (clinical measurement) and other favorable pathological characteristics, such as superficial stromal invasion (≤10 mm or 5 mm) and no lymphovascular space involvement (LVSI), the rates of parametrial invasion (PI) and lymph node metastasis (LNM) have been reported to be no greater than 1% and 2.4%, respectively, which are significantly reduced compared with the rates in patients with larger tumors^4^,^5^,^6^,^7^,^8^,^9^,^10^,^11^,^12^,^13^,^14^. Superficial stromal invasion and the absence of LVSI are potentially favorable factors for PI^15^,^16^ in stage Ib1 patients and for LNM in stage Ia2 to IIa1 patients^17^. Patients with these low-risk factors may benefit from curtailed surgery, such as simple hysterectomy and local conization, and may not require lymphadenectomy and other procedures. A reliable method for determining the relevant pathological parameters in these patients before surgery is necessary but has not yet been established. Conization procedures, such as cold knife cone (CKC) biopsy or loop electrosurgical excision procedure (LEEP), have been applied for the diagnosis and treatment of pre- and micro-invasive cervical cancers. Several previous reports have suggested the potential roles of conization procedures in evaluations of pathologic variables, which could be used as selection criteria for less radical surgery^15^,^16^,^18^ and even lymphadenectomy omission^17^ in patients with early-stage cervical cancer. However, the accuracy of these procedures for predicting pathologic variables in RH specimens is not yet well established.

Thus, the current study focused exclusively on patients with Ia2–Ib1 (≤2 cm) cervical cancer who had undergone a conization procedure before RH. We aimed to evaluate the predictive accuracy of conization pathological parameters for determining the corresponding parameters in RH specimens. The role of conization procedures in identification of a subset of patients who may be suitable for conservative treatment in future clinical trials is also discussed.

## Materials and Methods

The medical records of women with stage Ia2 to Ib1 cervical cancer who were treated at 4 hospitals from January 2003 to January 2014 were reviewed. These hospitals included Peking Union Medical College Hospital, Affiliated Hospital of Medical College Qingdao University, Second Hospital of Hebei Medical University, and Beijing Chao-Yang Hospital (affiliated with China Capital Medical University). The inclusion criteria were as follows: (1) the presence of FIGO stage Ia2 to Ib1 cervical cancer and receipt of RH and pelvic lymph node (LN) dissection; (2) a tumor size of ≤2 cm evaluated preoperatively by pelvic examination combined with imaging analysis, such as ultrasonic inspection, computed tomography (CT) and/or magnetic resonance imaging (MRI); and (3) receipt of CKC biopsy or LEEP before RH. Patients were excluded if they received preoperative radiation or chemotherapy, and those with cervical stump cancer or without complete medical records were also excluded.

During the conization procedure, the cervix was swabbed with Lugol’s iodine solution before resection. This technique is useful for locating the ectocervical margins of the lesion. The cervical lesion was then excised en bloc for better interpretation of the orientation and margin status. Following LEEP resection, the base of the crater and resection margin (RM) were coagulated and cauterized by ball diathermy to achieve hemostasis. In CKC biopsy, stay sutures composed of 1–0 chromic catgut were placed at 9 and 3 o’clock to partially occlude the descending branch of the uterine artery, as well as for stabilization of the cervix. Suturing the base of the resulting crater and RM was more commonly performed to achieve hemostasis in CKC biopsy.

The demographic and clinico-pathological data were retrospectively reviewed. Tumor width was measured in a direction along the surface epithelium perpendicular to the stromal infiltration, and tumors were accordingly divided into either a ≤20 mm or >20 mm subgroup. The stromal invasion depth was measured perpendicularly from the basement membrane of the surface epithelium using an ocular micrometer. The degree of stromal invasion was classified as either deep or superficial based on the depth (>5 mm or ≤5 mm, respectively). LVSI was defined as the unequivocal presence of malignant cells in endothelial-lined spaces observed upon histological examination of the specimen. The marginal statuses of the conization specimens were evaluated to determine whether they had ectocervical, endocervical, or deep margins, and the results were interpreted as positive when any of the three margins were involved with carcinoma *in situ* or invasive cancer. If substantial residual tumor was noted in an RH specimen after previous conization, then the tumor width in the final RH histopathology was determined as the sum of the tumor widths of the cone biopsy and the RH specimen from the conization site. Similarly, the maximum depth of invasion in RH histopathology was determined as the sum of the invasion depths of the cone biopsy and the RH specimen from the conization site. PI was defined as either positive parametrial nodes or parametrial tissue that included direct tumor growth or spread via lymphovascular channels. Clinical disease staging was performed according to the 2009 FIGO staging system^19^. Two independent pathologists with extensive backgrounds in gynecologic pathology specifically reviewed all of the pathological slides for the purposes of this study and were blinded to the patients’ outcomes.

The patient records/information were anonymized and de-identified prior to analysis; therefore, consent was not necessary. The study protocol was approved by the ethics committees at Peking Union Medical College Hospital, the Affiliated Hospital of Medical College Qingdao University, the Second Hospital of Hebei Medical University, and Beijing Chao-Yang Hospital (affiliated with China Capital Medical University). This study was carried out in accordance with the ethical standards of the responsible committee on human experimentation (institutional and national) and with the Helsinki Declaration of 1975, as revised in 2008.

## Statistical analysis

All statistical analyses were performed using SAS® Version 9.2 (SAS Institute, Cary, NC). All tests were 2-sided, and P-values of ≤0.05 were considered statistically significant. Chi-squared tests and logistic regression analyses were performed to identify the high-risk factors associated with the presence of residual lesions in the hysterectomy specimens after the conization procedures. The sensitivity, specificity, positive predictive value (PPV), negative predictive value (NPV), and false negative rate (FNR) were calculated to evaluate the predictive accuracy of conization for determination of the final RH pathological diagnosis.

## Results

A total of 297 patients who met the inclusion criteria were included in this study (Table 1). The mean age at diagnosis was 41.4 ± 8.6 (range: 22–74) years. Premenopausal women accounted for 84.2% (250/297) of the patients. A total of 168 patients (56.6%) received laparotomy, and 129 (43.4%) underwent laparoscopic surgery. Further, 50 (16.8%) patients had stage Ia2 cancer, and 247 (83.2%) had stage Ib1 cancer. The tumor histologies were squamous cell carcinoma (SCC) in 279 (93.9%) patients, adenocarcinoma in 16 (5.4%), and adenosquamous carcinoma in 2 (0.7%). Grade 1, 2 and 3 tumors were detected in 79, 136 and 82 of the patients, respectively. Overall, LVSI was detected in 48 (16.2%) patients, and 4 (1.3%) had vaginal invasion. In addition, PI was identified in 2 patients (0.6%), including one with a positive left parametrial node. The mean number of LNs removed was 20.1 ± 8.0 per patient (range: 8–54). Fifteen women (5.1%) had LNM, with a total of 48 positive nodes.

CKC biopsy was performed in 257 (86.5%) patients, and the remaining patients (n = 40, 13.5%) underwent LEEP. Of these patients, 155 (52.2%) presented with invasion of the RM in the conization specimen, and 94 (60.6%) had residual disease in the hysterectomy specimen. In the remaining 142 (47.8%) patients with clear RMs in the conization specimen, 3 had a residual lesion in the hysterectomy specimen, including two with adenocarcinoma and one with SCC (Table 2). Thus, a total of 97 (32.7%) patients had residual lesions in the hysterectomy specimens.

**Figure 1:**
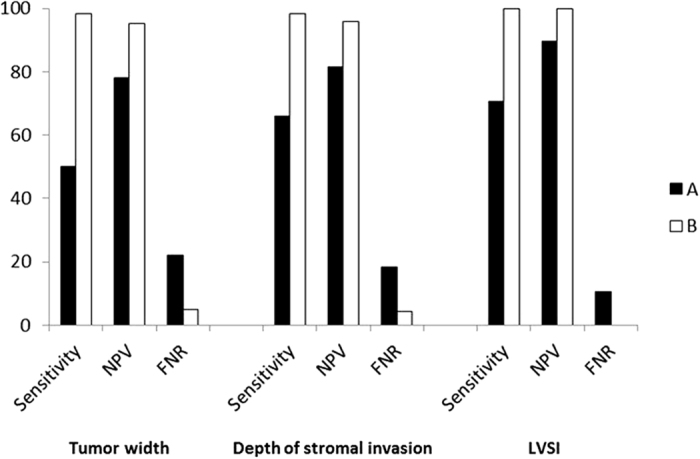
The sensitivity and NPV of the conization procedure for predicting pathological parameters in hysterectomy specimens using a simple prediction model (A) were far from satisfactory (tumor width: 50.0% and 78.0%, respectively; depth of stromal invasion: 66.1% and 81.6%; and LVSI: 70.5% and 89.5%, respectively). In contrast, the combined prediction model (B) was quite efficient, with the following results for sensitivity and NPV: tumor width: 98.2% and 95.2%, respectively; depth of stromal invasion: 98.4% and 95.8; and LVSI: 100% and 100%.

The sensitivity, specificity, PPV and NPV of conization for detecting residual disease in the hysterectomy specimens were 96.9%, 69.5%, 60.6% and 97.9%, respectively. Undoubtedly, the specificity and PPV of conization for predicting tumor width, stromal invasion depth and LVSI in the final RH pathology analysis were all 100%. In addition, the sensitivity and NPV of this procedure in cases with clear margins for predicting these parameters were satisfactory (width: 87.5% and 99.3%; depth: 90.5% and 98.4%; LVSI: 100% and 100%, respectively). The FNRs were as low as 0.7%, 1.6% and 0, respectively. However, these parameters of conization with margin invasion increased the concern of gynecologic oncologists. Based on these observations, a clinical decision was made for conservative surgery. The sensitivity and NPV of conization in the margin-invaded subgroup were 50.0% and 78. 0%, respectively, for predicting tumor width (≤2 cm vs. >2 cm) in the final RH pathology analysis, as determined using conization specimens from 28 cases with a tumor width of ≤2 cm but with a width of >2 cm in the RH specimens (Table 3 and Fig. 1). Moreover, conization failed to predict deep stromal invasion in 21 patients in the margin-invaded subgroup, which was subsequently confirmed in the final RH pathology analysis. The sensitivity and NPV of conization were 66.1% and 81.6%, respectively, for predicting tumor depth (≤5 mm vs. >5 mm) in the final RH pathology analysis, and the FNR was 18.4%. Conization failed to predict LVSI in 13 cases in the margin-invaded subgroup, and these findings were later confirmed in the hysterectomy specimens. Thus, the sensitivity and NPV of conization with margin invasion for predicting LVSI in the final RH pathology analysis were 70.5% and 89.5%, respectively. Hence, a simple prediction model was constructed with a single conization variable in the margin-invaded subgroup to identify the corresponding variable in the final RH pathology analysis, but this model was not sufficiently effective.

Residual disease in the hysterectomy specimens was a major factor that was negatively associated with the sensitivity and NPV of conization. The pathological parameters in the conization specimens that were significantly associated with the presence of residual disease in the hysterectomy specimens included tumor stage, tumor width, stromal invasion depth, LVSI, and the involvement of endocervical or deep RMs, as shown by univariate analyses (P = 0.016, 0.001, <0.001, = 0.010, <0.001, and <0.001, respectively; Table 4). LEEP was not a notable predictor of residual disease in the hysterectomy specimens compared with CKC (33.3% vs. 32.3%, P = 0.734). Involvement of endocervical or deep RMs was found to be an independent risk factor in multivariate analysis (P < 0.001, = 0.003, respectively). The residual tumor rate was only 4.2% (7/167) in the women with clear endocervical and deep RMs, while it was 69.2% (90/130) in those with positive endocervical and/or deep RMs. Thus, these two factors were added to the prediction model, and its predictive accuracy was then re-evaluated.

In the combined prediction model, a tumor width of >20 mm in the final RH pathology analysis was predicted by a tumor width of >20 mm or involvement of endocervical or deep RMs in the conization specimens, and the sensitivity and NPV of conization with margin invasion were as high as 98.2% and 95.2% (both P < 0.001), respectively. Similarly, the sensitivity (98.4%) and NPV (95.8%) of conization for predicting deep stromal invasion (>5 mm) was significantly increased (both P < 0.001) in the margin-invaded subgroup when it was predicted by deep stromal invasion (>5 mm) or the involvement of endocervical or deep RMs in the conization specimens. The corresponding sensitivity and NPV of the new prediction model for predicting LVSI in the final RH pathology analysis in the invaded-margin subgroup were both 100% (both P < 0.001). In addition, the FNRs of the combined prediction model for identifying these parameters were significantly decreased (4.8%, 4.2%, and 0, respectively).

## Discussion

Accumulating retrospective evidence suggests that conservative surgery is potentially feasible for patients with stage Ia2 to b1 cervical cancer who have a specific set of tumor characteristics. In addition to the histological type and tumor grade, the tumor size, depth of invasion, and presence of LVSI are the most commonly used predictors of patients suitable for conservative surgery. In a multicenter retrospective study^14^, we also revealed that a tumor size of ≤2 cm was an independent predictor of vaginal invasion, PI, and uterine isthmus invasion. Grade 2/3 disease, deep stromal invasion, and LVSI were found to be independently associated with LNM.

Based on the results of these previous reports, we hypothesized that the preoperative determination of tumor size and invasion depth, as well as the presence of LVSI, in CKC biopsy or LEEP specimens, may influence the decision to offer RH and lymphadenectomy to cervical cancer patients who appear to be at low risk of PI and LNM. The selection criteria vary in different patient populations and with the use of different statistical screening models. Further large-scale clinical trials are warranted to validate their safety and practicality for identifying a patient subset that is potentially suitable for conservative surgery. Thus, we aimed to elucidate the relationships of conization pathological parameters with the final histopathology findings from hysterectomy specimens, with the goal of preoperatively identifying these predictors. However, residual disease is present in over half (60.6%) of hysterectomy specimens obtained after conization with positive margins. Our data indicated that the conization procedures had poor sensitivity and a low NPV for determining the final RH pathological variables if the margin status of the conization specimen was not included in the prediction model. Bidus *et al*.^20^ have also revealed that the NPV of conization is insufficient for LVSI prediction in hysterectomy specimens from cervical cancer patients if subsequent hysterectomy specimens have residual cancer. Further, Kim *et al*.^21^ have reported that the NPV of LEEP is low for the prediction of LVSI and stromal invasion depth in final hysterectomy specimens, which is in agreement with our findings.

The presence of residual disease in hysterectomy specimens is a major factor that is negatively associated with the sensitivity and NPV of conization. Suri *et al*.^22^ have found that positive margins in conization specimens predict the presence of residual disease in RH specimens of women with stage Ia2 cervical cancer. Further, Kim *et al*.^21^ have demonstrated that involvement of endocervical or deep RMs is an independent predictor of residual disease in hysterectomy specimens. However, neither of these two groups further evaluated the sensitivity or NPV of conization after incorporating endocervical and deep RMs in the prediction model. The present study confirmed that positive endocervical or deep RMs in conization specimens were an independent risk factor for residual disease in hysterectomy specimens. Our results showed that the rate of residual tumor was only 4.2% in the women with clear endocervical and deep RMs compared with 69.2% in those with positive endocervical and/or deep RMs. When a tumor width of >20 mm in the final RH pathology analysis was predicted by a tumor width of >20 mm or involvement of the endocervical or deep RMs in the conization specimens, the sensitivity and NPV of conization with margin invasion increased to 98.2% and 95.2%, respectively, with an FNR of 4.8%. Further, when deep stromal invasion (>5 mm) in the final samples was predicted by deep stromal invasion (>5 mm) or involvement of the endocervical or deep RMs in the conization specimens, the sensitivity and NPV of conization were as high as 98.4% and 95.8%, respectively, and the FNR was as low as 4.2%. In addition, the corresponding sensitivity and NPV of this prediction model for predicting LVSI in the final RH pathology analysis were both 100%, respectively, and the FNR was zero. Thus, conization variables and the endocervical and deep RM statuses are relatively effective predictors of the final RH pathological parameters. This new prediction model appeared to be feasible for identifying a subset of patients with stage Ia2 to Ib1 (≤2 cm) cervical cancer with low-risk factors before surgery who may be suitable for conservative treatment in future clinical trials.

Two conization methods, including CKC biopsy and LEEP, were applied in the present study. It has been argued that the tissue margins in a LEEP biopsy may contain significant thermal artifacts that can interfere with pathological assessment of biopsy margins^23^,^24^. CKC biopsy has been used as a conventional technique for the diagnosis and treatment of high-grade cervical intraepithelial neoplasia and for the diagnosis of early-stage cervical cancer. However, LEEP has become more common in recent years. The accepted advantages of LEEP include the avoidance of general anesthesia, provision of treatment in an outpatient setting, decreased morbidity, and reduced rates of obstetric complications, all of which have significant cost benefits^25^,^26^,^27^,^28^,^29^,^30^. In addition, the present analysis revealed that the residual disease risk of LEEP was not significantly increased compared with that of CKC, in agreement with previous reports^25^,^26^,^31^,^32^,^33^,^34^. Taken together, these findings could potentially alter physicians’ preferences for LEEP in clinical practice. In our opinion, CKC biopsy should be performed conservatively, at least for patients who have a desire to preserve fertility.

Several preoperative studies have preliminarily demonstrated that conization plus pelvic lymphadenectomy appears to be feasible for the conservative management of early-stage cervical cancer and that it results in a low risk of relapse, provided that the patient presents with certain “favorable” tumor characteristics^35^,^36^,^37^. However, all of these analyses have been limited by evaluation of a very small sample size and lack of randomized controls. In an ongoing multicenter phase III trial (NCT02368574), we are aiming to evaluate the clinical benefits of parametrectomy omission in stage I a2 to I b1 patients with a tumor size of <2 cm and superficial stromal invasion. Conization (as well as MRI examination) is used in preoperative evaluations during patient enrollment. The predictive accuracy of the conization procedure for identifying pathological parameters in hysterectomy specimens is being further evaluated in this trial.

A strength of the present study is its multi-institutional nature and relatively large sample size. The CKC biopsy and LEEP specimens were obtained by multiple independent practitioners. An additional strength is the relative completeness of the pathological reports. These strengths enabled us to perform robust analyses to identify the important factors affecting residual disease in RH specimens and to consequently evaluate the sensitivity and NPV of conization for predicting the final RH pathological parameters. The major limitation of this analysis is its retrospective nature. The preoperative conization procedure was mainly performed on patients with small tumors and on those with potentially favorable factors, which may have resulted in selection bias to some extent. However, this cohort of patients is the main focus of studies on conservative surgery.

Consequently, the involvement of endocervical or deep RMs in conization specimens is an independent predictor of residual disease in hysterectomy specimens from patients with stage Ia2 or Ib1 (≤2 cm) cervical cancer. RH pathological parameters, such as tumor size, stromal invasion depth, and the presence of LVSI, can all be reliably predicted by analyses of the corresponding conization variables and the endocervical and deep RM statuses. Thus, patients exhibiting specific low-risk factors in their conization specimens can be spared from RH surgery and/or LN dissection.

## Additional Information

**How to cite this article**: Bai, H. *et al*. Accuracy of conization procedure for predicting pathological parameters of radical hysterectomy in stage Ia2–Ib1 (≤2 cm) cervical cancer. *Sci. Rep.* **6**, 25992; doi: 10.1038/srep25992 (2016).

## Figures and Tables

**Table 1:** Clinicopathologic characteristics of the 297 patients.

Parameter	Number of patients	Percent (%)
Age, mean (range), yrs: 41.4 ± 8.6 (22–74)		
≤45	207	69.7
>45	90	30.3
Tumor size^a^, mean (range), cm: 1.2 ± 0.6 (0.2–2.0)		
Stage		
Ia2	50	16.8
Ib1	247	83.2
Histology		
SCC^b^	279	93.9
AD^c^	16	5.4
ASC^d^	2	0.7
Grade		
1	79	26.6
2	136	45.8
3	82	27.6
Conization parameter		
Conization		
LEEP^e^	40	13.5
CKC^f^	257	86.5
Tumor width, mean (range), mm: 12.98 ± 7.7 (0.2–4.0)		
≤20 mm	262	88.2
>20 mm	35	11.8
Depth of stromal invasion, mean (range), mm: 4.1 ± 2.3 (1.0–13.0)		
≤5 mm	237	79.8
>5 mm	60	20.2
LVSI^g^	47	15.8
Positive RM^h^	155	52.2
Ectocervical RM	48	16.2
Endocervical RM	103	34.7
Deep RM	51	17.2
RH^i^ parameter		
Surgical approach		
Laparotomy	168	56.6
Laparoscopy	129	43.4
Residual disease	97	32.7
Carcinoma *in situ*	23	23.7
Invasive cancer	74	76.2
Tumor width, mean (range), mm: 14.0 ± 8.4 (0.2–4.0)		
≤20 mm	233	78.5
>20 mm	64	21.5
Depth of stromal invasion, mean (range), mm: 4.6 ± 2.4 (1–15)		
≤5 mm	215	724
>5 mm	82	27.6
LVSI	48	16.2
Vaginal invasion	4	1.3
Parametrial invasion	2	0.6
LNM^j^	15	5.1
Pelvic	13	4.4
Para-aortic	2	0.7
Positive RM	0	0

^a^Clinically measurable tumors only; ^b^Squamous cell carcinoma; ^c^Adenocarcinoma; ^d^Adenosquamous carcinoma; ^e^Cold knife cone; ^f^Loop electrosurgical excision procedure; ^g^Lymphovascular space involvement; ^h^Resection margin; ^i^Radical hysterectomy; ^j^Lymph node metastasis.

**Table 2:** Clinicopathologic characteristics of the 3 patients with residual lesions in the hysterectomy specimen but with clear resection margins in the conization specimens.

Patient	Age	Stage	Histology	Grade	Conization parameter				RH parameter			
					Tumor width (mm)	Depth of stromal invasion (mm)	LVSI	RM	Residual disease	Tumor width (mm)	Depth of stromal invasion (mm)	LVSI
1	48	Ib1	AD	1	29	5	−	−	+	33	7	−
2	56	Ib1	SCC	2	5	3	−	−	+	7	4	−
3	47	Ib1	AD	3	15	7	+	−	+	29	9	+

**Table 3:** Accuracy of the conization procedure for predicting the final RH pathological diagnosis in the margin-invaded subgroup.

Parameter	Model	Sensitivity (%) (95% CI^a^)	P value^b^	NPV^c^ (%) (95% CI^a^)	P value^b^	FNR ^d^ (%) (95% CI^a^)	P value^b^
Tumor width	A^e^	50.0 (36.9–63.1)	<0.001	78.0 (70.7–85.2)	<0.001	22 (15.2–30.3)	<0.001
	B^f^	98.2 (90.5–99.9)		95.2 (76.2–99.9)		4.8 (0–13.9)	
Depth of stromal invasion	A^e^	66.1 (53.0–77.3)	<0.001	81.6 (73.2–88.2)	<0.001	18.4 (11.8–26.8)	<0.001
	B^f^	98.4 (95.2–100)		95.8 (87.8–100)		4.2 (0–12.2)	
LVSI	A^e^	70.5 (54.8–83.2)	<0.001	89.5 (82.7–94.3)	<0.001	10.5 (5.7–17.3)	<0.001
	B^f^	100 (92.0–87.9)		100 (83.9–100)		0 (0–16)	

^a^Credibility interval; ^b^McNemar chi-square test; ^c^Negative predictive value; ^d^False negative rate; ^e^Simple prediction model; ^f^Combined prediction model (RH pathological parameters were predicted with the corresponding conization variables combined with the endocervical and deep resection margin statuses of conization.).

**Table 4:** Conization clinico-pathological parameters associated with presence of residual disease in the hysterectomy specimens.

Parameter	Residual Disease		p value^a^	p value^b^
	+	−		
Age				
≤45	64	143	0.332	
>45	33	57		
Conization				
LEEP	14	26	0.734	
CKC	83	174		
Stage				
Ia2	9	41	0.016	
Ib1	88	159		
Histology				
SCC	88	191	0.106	
AD + ASC	9	9		
Grade				
1	23	56	0.433	
2 + 3	74	144		
Tumor width				
≤20 mm	77	185	0.001	
>20 mm	20	15		
Depth of invasion				
≤5 mm	65	172	<0.001	
>5 mm	32	28		
LVSI				
+	23	24	0.010	
−	74	176		
Endocervical RM				
+	73	30	<0.001	<0.001
−	24	170		
Ectocervical RM				
+	17	31	0.657	
−	80	169		
Deep RM				
+	28	23	<0.001	0.003
−	69	177		

^a^Chi-square test; ^b^Logistic regression analysis.
